# Accuracy in Microanalysis by Electron Energy-Loss Spectroscopy

**DOI:** 10.6028/jres.093.082

**Published:** 1988-06-01

**Authors:** Ray F. Egerton

**Affiliations:** Department of Materials Science and Engineering, State University of New York, Stony Brook, NY 11794-2275

The transmission electron microscope can focus electrons onto a small region of a specimen, typically 1 nm to 1 µm in diameter. If the specimen is suitably thin (preferably <100 nm) and the transmitted electrons enter a high-resolution electron spectrometer, an electron energy-loss spectrum is produced. This spectrum (fig. 1) contains a zero-loss peak, representing elastic scattering, one or more peaks in the 4–40 eV range (due to inelastic scattering from outer-shell electrons) and, at higher energy loss and lower intensity, characteristic edges due to ionization of inner atomic shells. These latter features are used in elemental microanalysis, usually by fitting a background in front of each edge and measuring the area *I*_c_ over an energy range Δ beyond each edge; see figure 1. The number of atoms (*N* per unit specimen area) of a particular element can be obtained from [1]:

$$N≃I_{c}/GI_{l}\sigma_{c})$$ (1)

The factor *G* makes allowance for any increase in detector gain between recording the low-loss region (area *I*_l_) and the ionization edges; *σ*_c_ is a cross section for inner-shell scattering over the appropriate range of energy loss, which can be calculated from atomic theory or obtained experimentally. Energy-loss spectroscopy is therefore capable of providing absolute, standardless elemental analysis, although in practice it is usually the ratio of two elements which is of interest, in which case the quantities *G* and *I*_l_ cancel and need not be measured.

Energy-loss spectroscopy has been used to identify quantities of less than 10^−20^ g and concentrations of less than 100 ppm of elements such as phosphorus and calcium in an organic matrix [2,3]. However, the accuracy of quantitative analysis, using eq (1), is often no better than 20%. The main sources of error, and possibilities for their removal, are discussed below.

## Background Subtraction

The pre-edge background is usually taken to be a power law: *AE*^−^*^r^* where *E* represents energy loss, *A* and *r* being found by least-squares fitting over a pre-edge region. Particularly for lower-energy edges where the background is relatively high, alternative fitting functions or procedures have been used to reduce systematic errors [2,4–6]. Also because the background must be extrapolated rather than interpolated, statistical errors tend to be high [6]. Both types of error could be reduced by fitting over both the pre-edge and a post threshold region [7,8]. Where the elemental concentration is low, the use of digital filters [9] and differential-mode spectrum recording [2] is being developed.

## Effect of Elastic Scattering

**Figure 2:**
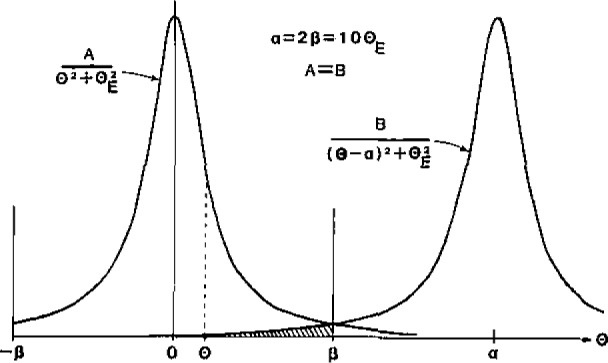
Angular distributions of inelastic scattering around the undiffracted beam and around a beam diffracted through an angle *α*. The shaded area represents the contribution from the diffracted beam to the intensity collected by an on-axis aperture of semi-angle *β*.

Equation (1) would be exact if inner-shell excitation were the only mode of scattering or if all scattered electrons contributed to the energy-loss spectrum. In practice, the spectrometer collects scattering only up to some angle *β*, and this fact is taken into account to first order by using a cross section *σ*_c_ (*β*,Δ) evaluated over the appropriate angular range. However, to reduce the background contribution from plural scattering, *β* is usually chosen to be in the range 3–15 mrad, and as a result most of the *elastically* scattered electrons are excluded from the spectrometer. With a crystalline specimen, diffracted beams contribute additional intensity to *I*_c_, in the form of plural (elastic + inner-shell) scattering [1,10], causing *N* to be overestimated. The systematic error involved depends on the edge energy (which determines the angular width *θ_E_* of the inner-shell scattering) and the specimen thickness and orientation (which determine the intensity of the diffracted beams); see figure 2. It is therefore not surprising to find that elemental ratios measured from test samples become inaccurate for thicker specimens or under strongly diffracting conditions [1,6,11]. Efforts are continuing to find a simple method of correcting for the diffracted electrons. In an amorphous material, the equivalent errors should be much reduced [1].

## Accuracy of Inner-Shell Cross Sections

The accuracy of absolute quantification is clearly dependent on the accuracy with which *σ_c_* is known; likewise, the accuracy of elemental ratios will depend on the *relative* accuracy of the inner-shell cross sections. *K*-shell cross sections can be calculated rapidly (on-line) by use of a hydrogenic approximation, probably to an accuracy of around 10% [12]. Hydrogenic *L*-shell cross sections require some empirical correction, originally based on photoabsorption data [13].

Hartree-Slater or Dirac-Slater calculations can be carried out for all atomic shells [14] and should be more accurate, though more time-consuming. Within the last year or two, systematic experimental determinations of *K*-, *L*-, and *M*-shell cross-section ratios have been made for the ionization edges of most interest [15,16]. As a result, correction parameters used in the hydrogenic *L*-shell program have been modified [6] and further refinement may be desirable. A parameterization scheme for all edges of practical importance is also being investigated since the measured cross-section ratios (“*k*-factors”) apply only for given values of *β*, Δ and incident-electron energy.

## Lens-Aberration Errors

Spherical and chromatic aberrations of any electron lenses between the specimen and spectrometer can cause a loss of energy resolution, spatial resolution and collection efficiency, all of which could lead to analysis errors, dependent on the values of *β*, edge energy, spatial resolution (e.g., incident-beam diameter) and the type of electron-optic coupling [6]. Severe effects have been reported in cases where one of the post-spectrometer lenses is operating with an object distance close to its focal length [17]. More work needs to be done to assess the performance of conventional microscope lenses at high energy loss.

## Radiation Damage

The electron beam used for microanalysis can cause both structural damage and chemical change (mass loss) within the analyzed region, leading to errors in elemental ratios. With a high-brightness electron source, this problem is observable even with inorganic specimens [18]. Use of a liquid-nitrogen stage may reduce the radiation sensitivity, particularly of organic specimens. Parallel-recording spectrometers, now commercially available, will greatly increase the signal-collection efficiency and reduce the electron exposure required to obtain an acceptably noise-free spectrum. In fact, the radiation-damage problem is not so severe as in x-ray emission spectroscopy, where the collection efficiency and fluorescence yield (for light elements) are both much lower.

## Figures and Tables

**Figure 1:**
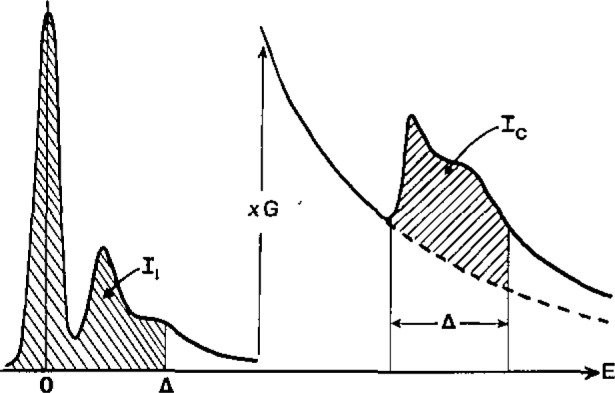
Schematic energy-loss spectrum with a gain increment *G* between the low-loss and high-loss regions.
